# Temperature Drops and the Onset of Severe Avian Influenza A H5N1 Virus Outbreaks

**DOI:** 10.1371/journal.pone.0000191

**Published:** 2007-02-07

**Authors:** Chung-Ming Liu, Shu-Hua Lin, Ying-Chen Chen, Katherine Chun-Min Lin, Tsung-Shu Joseph Wu, Chwan-Chuen King

**Affiliations:** 1 Global Change Research Center, National Taiwan University, Taipei, Taiwan; 2 Department of Atmospheric Sciences, College of Science, National Taiwan University, Taipei, Taiwan; 3 Department of Public Health, College of Public Health, National Taiwan University, Taipei, Taiwan; 4 Institute of Epidemiology, College of Public Health, National Taiwan University, Taipei, Taiwan; Institute of Human Virology, United States of America

## Abstract

Global influenza surveillance is one of the most effective strategies for containing outbreaks and preparing for a possible pandemic influenza. Since the end of 2003, highly pathogenic avian influenza viruses (HPAI) H5N1 have caused many outbreaks in poultries and wild birds from East Asia and have spread to at least 48 countries. For such a fast and wide-spreading virulent pathogen, prediction based on changes of micro- and macro-environment has rarely been evaluated. In this study, we are developing a new climatic approach by investigating the conditions that occurred before the H5N1 avian influenza outbreaks for early predicting future HPAI outbreaks and preventing pandemic disasters. The results show a temperature drop shortly before these outbreaks in birds in each of the Eurasian regions stricken in 2005 and 2006. Dust storms, like those that struck near China's Lake Qinghai around May 4, 2005, exacerbated the spread of this HPAI H5N1 virus, causing the deaths of a record number of wild birds and triggering the subsequent spread of H5N1. Weather monitoring could play an important role in the early warning of outbreaks of this potentially dangerous virus.

## Introduction

In most studies, the central factor thought to contribute to the unprecedented spread of H5N1 or novel avian influenza A viruses (AIV) has been the interaction between migrating waterfowl and domestic poultry in Eurasia [1]–[2], though the poultry and wild bird trade has also been implicated [1], [3]–[4]. Although wild birds are a natural reservoir of low pathogenic avian influenza (LPAI), we do not know if wild birds are a permanent reservoir of highly pathogenic avian influenza (HPAI) or how a LPAI virus transforms into a HPAI virus, one that poses a great threat to animal health and public health [3], [5]. With regard to prevalence, samples collected in Europe between 2005 and 2006 by the Food and Agricultural Organization (FAO) of the United Nations showed only the H5N1 in dead wild birds, not in live ones [6], though their sample size and sampling techniques still have to be improved [7]. Meanwhile, all countries that have experienced HPAI H5N1 outbreaks have taken stringent measures to try to curb the spread of this virus [8]. Still, dead birds carrying the H5N1 virus continue to be reported in most of Asia and Europe. What has both triggered the sudden changes occurring in many outbreaks and initiated its global spread remains unclear.

In this study, we look beyond virology to other factors, namely changes in weather patterns that might contribute to the increase of outbreak occurrence and spread of the fatal pathogen HPAI H5N1 among live birds. We hypothesized that H5N1 viruses, which are carried by live birds, would not be activated randomly, instead, they would be automatically and predictably elicited and imposed by acute environmental health stress like sudden cold air masses.

## Methods

The study method started by searching for each AIV HPAI H5N1 outbreak date first, followed by analyzing the synoptic and local meteorological data collected from the website of the United States National Climate Data Center (NCDC). Since some outbreak areas were not associated with a World Meteorological Organization (WMO) registered weather station, and many outbreaks were happening in a close range within a short period of time, a total of twenty-seven representative cases (Table 1) are analyzed here.

**Table 1:** Selected HPAI H5N1 virus outbreaks and the minimum temperatures and dates at nearby WMO (World Meteorological Organization) stations.

Group		Reported date of each outbreak year/month/date	Region	WMO station no.	Studied period	Latitude	Longitude	Minimum temperature (date)	Primary species of infected birds or poultry involved
I (2005/5)	a	2005/05/04	Qinghai, China	527540	5/2∼5/11	37.3N	100.1E	−8°C (5/5)	bar-headed geese, whooper swans
II (2005/7 ∼ 10)	a	2005/07/23	Suzdalka, Russia	297260	7/17∼7/26	54.4N	82.0E	11.1°C (7/19)	wild waterfowl
	b	2005/08/20	Almaty, Russia	369110	8/12∼8/21	42.8N	75.3E	12.9°C (8/14)	wild ducks
	c	2005/10/01	Turkey	171150	9/26∼10/5	40.3N	28.0E	10.0°C (9/28)	turkeys
	d	2005/10/07	Romania	153600	10/5∼10/14	45.2N	29.7E	12.2°C (10/7)	swans, hens and ducks
	e	2005/10/14	Tula, Russia	277190	10/8∼10/17	54.2N	37.6E	−0.2°C (10/10)	ducks; muscovy ducks; chickens; geese; turkeys
III (2005/10 ∼ 11)	a	2005/10/14	Nei Mongol, China	534630	10/11∼10/20	40.8N	111.7E	−6.0°C (10/13)	chickens and ducks
	b	2005/10/26	Liaoning, China	543424	10/19∼10/28	41.6N	123.4E	−6.0°C (10/21)	chickens; magpies and wild birds
	c	2005/11/09	Xinjiang, China	514630	11/3∼11/12	43.8N	87.6E	−2.1°C (11/5)	chickens and ducks
	d	2005/11/10	Shanxi, China	538630	11/6∼11/15	37.0N	111.9E	−2.6°C (11/8)	chickens and geese
IV (2005/11∼2006/1)	a	2005/11/25	Turkey	170980	11/22∼12/1	40.6N	43.1E	−11.1°C(11/24)	chickens
	b	2005/12/22	Ukraine	331770	12/18∼12/27	50.8N	24.3E	−15.6°C(12/20)	chickens
	c	2005/12/30	Turkey	171600	12/24∼1/2	39.2N	34.2E	−7.3°C (12/26)	chickens, geese, turkeys and ducks
	d	2006/01/13	Ukraine	339020	1/11∼1/20	46.6N	32.6E	−9.1°C (1/13)	chickens, geese, turkeys and ducks
	e	2006/01/26	Turkey	172020	1/20∼1/29	38.6N	39.3E	−14.0°C (1/22)	chickens, geese, turkeys, ducks and pigeon
V (2006/1 ∼ 3)	a	2006/01/30	Greece	166220	1/24∼2/2	40.5N	23.0E	−8.0°C (1/26)	swans
	b	2006/02/08	Germany	101720	2/4∼2/13	53.9N	12.3E	−9.0°C (2/6)	cat and wild birds
	c	2006/02/13	France	74810	2/10–2/19	45.7N	5.1E	−4.7°C (2/12)	turkeys
	d	2006/03/01	Poland	123100	2/25∼3/6	52.4N	14.6E	−10.0°C (2/27)	wild swans
	e	2006/03/10	Switzerland	66790	3/6∼3/15	47.5N	8.9E	−10.5°C (3/8)	tufted duck and common coot
VI (2006/2 ∼ 3)	a	2006/02/20	Egypt	624030	2/16∼2/25	26.2N	32.8E	6.0°C (2/18)	poultry flocks (chickens, turkeys, geese, ducks, breeders, broilers, layers, pigeons and peacocks) and migratory birds
	b	2006/03/14	Egypt	623180	3/9∼3/18	31.2N	30.0E	5.0°C (3/11)	poultry flocks (chickens, turkeys, geese, ducks, breeders, broilers, layers, pigeons and peacocks) and migratory birds
	c	2006/03/16	Egypt	624400	3/9∼3/18	30.6N	32.3E	6.4°C (3/11)	poultry flocks (chickens, turkeys, geese, ducks, breeders, broilers, layers, pigeons and peacocks) and migratory birds
VII (2006/4 ∼ 6)	a	2006/04/15	Qinghai, China	527540	4/13∼4/22	37.3N	100.1E	−9.8°C (4/15)	bar-headed geese
	b	2006/05/21	Qinghai, China	560460	5/19∼5/28	33.8N	99.7E	−4.4°C (5/21)	bar-headed goose, Tadorna ferruginea.
	c	2006/05/26	Omsk, Russia	286980	5/24∼6/2	55.1N	73.38E	6.0°C (5/26)	hens
	d	2006/06/15	Tomsk, Russia	294300	6/13∼6/22	56.5N	84.91E	6.4°C (6/15)	poultry and pigeons

## Results

### Outbreak at Lake Qinghai

In May and June 2005, the HPAI H5N1 virus killed an unprecedented six thousand bar-headed geese near Lake Qinghai in western China [7], [9], which was later linked to outbreaks in Eurasia. While many aspects of avian influenza have been discussed, no study has considered the relationship among these events, the drop of temperature (down to −8°C), and the strong dust storms that occurred in the region during May 2–4, 2005 (Fig. S1 in Supporting information), the period in which the massive death**s** of wild birds started.

**Figure 1:**
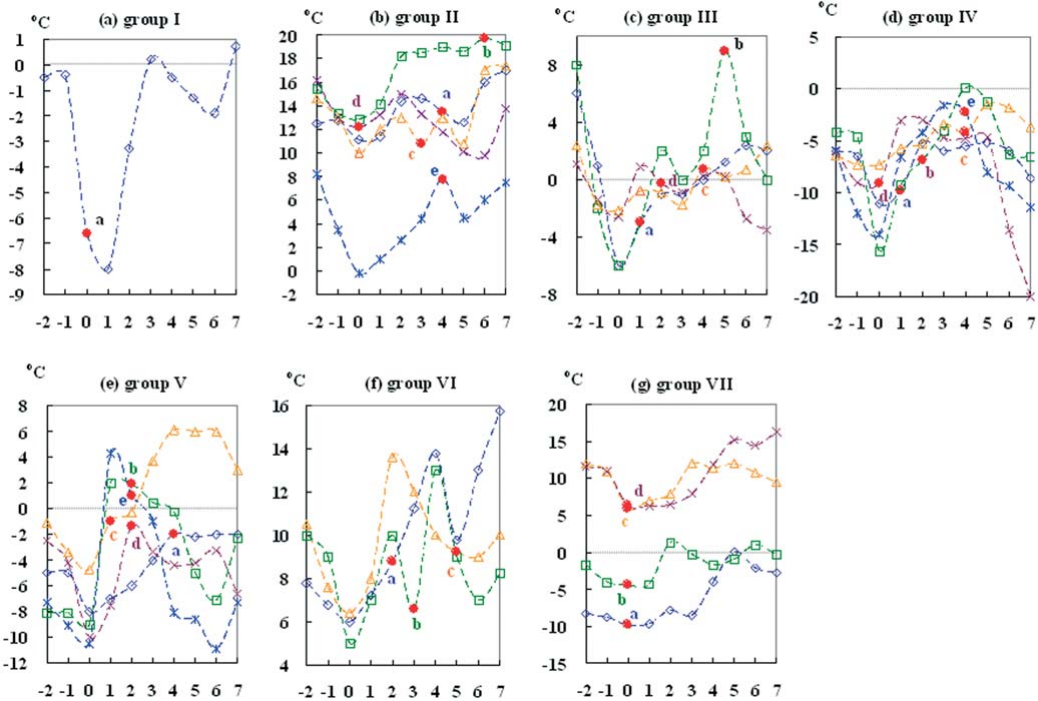
Variation of daily minimum temperatures at WMO stations near H5N1 virus outbreak areas. Each group and selected avian influenza H5N1 outbreaks events are listed in Table 1. Day 0 is the day with the lowest daily minimum temperature recorded; while the red dot represents the date of an outbreak of AIV was reported. For Figure 1(a) the day zero time point of group I was chosen as the time of the outbreak, not the time of the lowest temperature, since the former happened earlier.

In early May 2005, dust storms struck a wide area stretching from Mongolia to most of northwestern China (Figs. S1, S2, Table S1). These storms were caused by a swift-moving Siberian cold air mass that had channeled through valleys where several deserts locate (Fig. S2). Lake Qinghai, the site of the bar-headed goose deaths, is located about 500km east of the center of the Tsaidam Desert, where such large dust storms are rare [10]. However, it was at this time that strong wind stirred up a significant amount of dust locally. The cold air mass brought with it rapid temperature drops between the third and fifth of May, 2005, as recorded on the northern border of Lake Qinghai (Fig. 1a). On May 3, 4 and 5, temperatures dropped steeply from −0.4°C to −6.6°C and −8°C. Birds gathering around the lake, having just survived a long journey from southern China, then faced two major threats, i.e. subzero degrees temperatures and a sky filled with dust particles, simultaneously. Over the course of two to three days, these extreme and rapidly fluctuating conditions could have caused the birds enough physiological stress to suppress their immune systems [11]–[12]. Such two or three days of successive environmental stress and subsequent immunosuppression may have allowed the H5N1 virus to proliferate more efficiently in birds already carrying the virus, thereby increasing viral load and HPAI pathogenicity. These events would have hastened the inter-species spread of the virus and the deaths of wild birds over the following two months.

### Outbreaks in Eurasia

Outbreaks of avian flu H5N1 in Europe came with similar changes in weather conditions. By tracking the dates and locations of HAPI H5N1 outbreaks in Eurasia between July 2005 and June 2006 and cross-referencing that information with meteorological data from local WMO stations, we observed a striking correlation. All of the 27 AIV H5N1 outbreaks listed in Table 1 coincided 100% with drops in temperature (Fig. 1). We then categorized these outbreaks into seven groups (I–VII) by geographical areas and dates of occurrence (Table 1), and found that outbreaks of AIV H5N1 occurred by 6 days later after “day 0”, a term used here to represent the day that lowest daily temperatures were reached (Fig. 1). Most were actually reported within the first three days after day 0, and some on day 0, itself. Below freezing temperatures, as low as −15.6°C, were recorded for four of the seven outbreak groups (I, III, IV and V); above freezing temperatures were recorded for the two other groups. Each of the outbreaks in these groups was officially announced after the discovery of deceased birds, which may mean that some of the outbreaks occurred earlier than they were officially reported. It, therefore, follows that practically all of the outbreaks of H5N1 we studied occurred during or immediately following the sudden and steep temperature drops.

Two of the outbreaks, marked by nearby dust storms, were peculiar to the May 4, 2005 and April 15, 2006 outbreaks at China's Qinghai Lake (in Groups I and VII, respectively). The dust storms combined with the quick temperature drops may have contributed to the spread of H5N1 in these areas.

After careful investigation of the composite plots of surface pressures, temperatures and wind flows on day 0 of each selected H5N1 outbreaks (Figs. S3, S4, S5, S6 and S7), the arrival of colder air from higher latitude region may be the main cause of the spread. Within two days, the daily minimum temperature can drop by 3 to 14°C, which varied by location and season. Outbreaks of H5N1 from Turkey to France between November 2005 and March 2006 (Groups IV and V) were found to have the most striking temperature drops.

### Arrival of cold air mass

Most of the H5N1 outbreaks were associated with a Siberian cold air mass dominating Eurasia (Figs. S3, S4, S5, S6 and S7). This anticyclonic high pressure system moved southeastwardly transporting colder air on the eastern side of the system. The colder air flowed south toward the H5N1 outbreak area even during summer in southern Siberia (outbreaks II-a (Fig. S3), VII-d (Fig. S7)). In winter and spring, such high pressure system moved fast and brought below-freezing temperatures, termed widely as “cold air outbreak” events. On a few occasions, a portion of the cold air mass was separated from the main body and either became trapped in Turkey (outbreaks II-c (Fig. S3), IV-c and IV-e (Fig. S4)) or moved south to Egypt (outbreaks VI-a and VI-b (Fig. S7)), with much warmer temperatures than those reported in the northern areas from where the mass originated.

The occurrence of each of the lowest minimum temperatures was associated with both an arrival of cold air mass and an efficient radiative cooling under clear night-time skies. These conditions were sometimes followed by bright sunshine and air flows from a warmer region, resulting in a subsequent quick increase in temperature. In the outbreaks V-b and V-e, the temperatures increased by 11°C to 15°C just one day after day 0. H5N1 outbreaks were reported the next day. After examining the distribution of all the HPAI H5N1 outbreaks that occurred across many regions in the three major continents, the outbreaks indeed were associated with the very similar weather patterns as we mentioned above. Apparently, these dramatic weather fluctuations physically challenged the already fatigued migrating birds, just before official reports of avian flu outbreaks. This increased environmental stress is further strongly supported by the facts that HPAI H5N1 virus was detected in dead wild birds in even the more remote regions (outbreak VII-b) without poultry farms or trading centers [13].

## Discussion

It should be noted that our findings apply to regions north of the 25°N. How far the minimum temperatures would drop in these regions depends on season, altitude and synoptic weather conditions. Therefore, more studies investigating the optimal weather conditions before the HPAI H5N1 outbreaks in each region are needed before weather can be used to better predict outbreaks of this virus.

In conclusion, our findings show that regional weather monitoring may serve as an integral part of a bird flu surveillance system. We strongly advocate using temperature monitoring along with active virological surveillance [14] to help predict potential H5N1 outbreaks and identify where they might occur. International collaboration on such an initiative is urgently needed to respond to the potential threats of pandemic avian influenza.

## Supporting Information

Figure S1 Surface observation of dust storms happened on May 2–4, 2005. Symbols are explained in Table S1 and are WMO (World Meteorological Organization) standardized weather observation symbols. On May 1st, local dust storms associated with previous weather system were noted over deserts D2 and D4 (marked in Fig. 2). Then the intrusion of Siberia cold air mass forced dust storms over desert D1 on May 2nd, later over deserts D4, D2 and D3 on May 3rd and 4th. The sequence of occurrence and the eventually wide-spreading on May 4th followed with the movement of cold air mass marked as solid purple lines in Fig. S2.
(0.16 MB PDF)

Figure S2 Locations of Altay mountains (M1), Tianshan Mountains(M2), Karakorum Mountains(M3), Kunlun Mountains(M4), Arjin Mountains(M5), Qilian Mountains(M6), Gurbantunggut desert(D1), Taklamakan desert(D2), Tsaidam desert(D3), Gobi desert(D4), Tsaidam Basin (B), Gangca city (O) and the Bird Mountain (△) at Qinghai Lake. Square mark (▭) indicates the area in Qinhgai, China where wild birds with H5N1 virus were reported in spring 2006. Solid purple lines indicate the movement of Siberia cold air mass which would trigger the development of dust storms.
(0.19 MB PDF)

Figure S3 Contour plots of sea level pressure, surface temperature and wind flow on selected day 0 of outbreak event I-a (2005/5/4) and event II-a (2005/7/19), II-b (2005/8/14), II-c (2005/9/28), II-d (2005/10/7), II-e (2005/10/10).
(0.29 MB PDF)

Figure S4 Contour plots of sea level pressure, surface temperature and wind flow on selected day 0 of outbreak event III-a (2005/10/13), III-b (2005/10/21), III-c (2005/11/5), III-d (2005/11/8).
(0.18 MB PDF)

Figure S5 Contour plots of sea level pressure, surface temperature and wind flow on selected day 0 of outbreak event IV-a (2005/11/24), IV-b (2005/12/20), IV-c (2005/12/26), IV-d (2006/1/13), IV-e (2006/1/22).
(0.27 MB PDF)

Figure S6 Contour plots of sea level pressure, surface temperature and wind flow on selected day 0 of outbreak event V-a (2006/1/26), V-b (2006/2/6), V-c (2006/2/12), V-d (2006/2/27), V-e (2006/3/8).
(0.27 MB PDF)

Figure S7 Contour plots of sea level pressure, surface temperature and wind flow on selected day 0 of outbreak event VI-a (2006/2/18), VI-b,c (2006/3/11) and VII-a (2006/4/15), VII-b (2006/5/21), VII-c (2006/5/26), VII-d (2006/6/15). Each plot is downloaded from NOAA CDC Interactive Plotting and Analysis Pages (http://www.cdc.noaa.gov/Composites/Day/) using NCEP reanalysis data. In each figure, a white dot or a red dot is marked to indicate the area where avian influenza broke out.
(0.30 MB PDF)

Table S1 Meaning of weather symbols shown in Fig. S1.
(0.10 MB PDF)
