# Supplementary material for: Temperature Drops and the Onset of Severe Avian Influenza A H5N1 Virus Outbreaks
Source: PLoS One. 2007 Feb 7;2(2):e191. doi: 10.1371/journal.pone.0000191 (PMC1794318; doi:10.1371/journal.pone.0000191)
Supplement: Figure S1 — Surface observation of dust storms happened on May 2–4, 2005. Symbols are explained in Table S1 and are WMO (World Meteorological Organization) standardized weather observation symbols. On May 1st, local dust storms associated with previous weather system were noted over deserts D2 and D4 (marked in Fig. 2). Then the intrusion of Siberia cold air mass forced dust storms over desert D1 on May 2nd, later over deserts D4, D2 and D3 on May 3rd and 4th. The sequence of occurrence and the eventually wide-spreading on May 4th followed with the movement of cold air mass marked as solid purple lines in Fig. S2. (0.16 MB PDF) [file pone.0000191.s001.pdf]

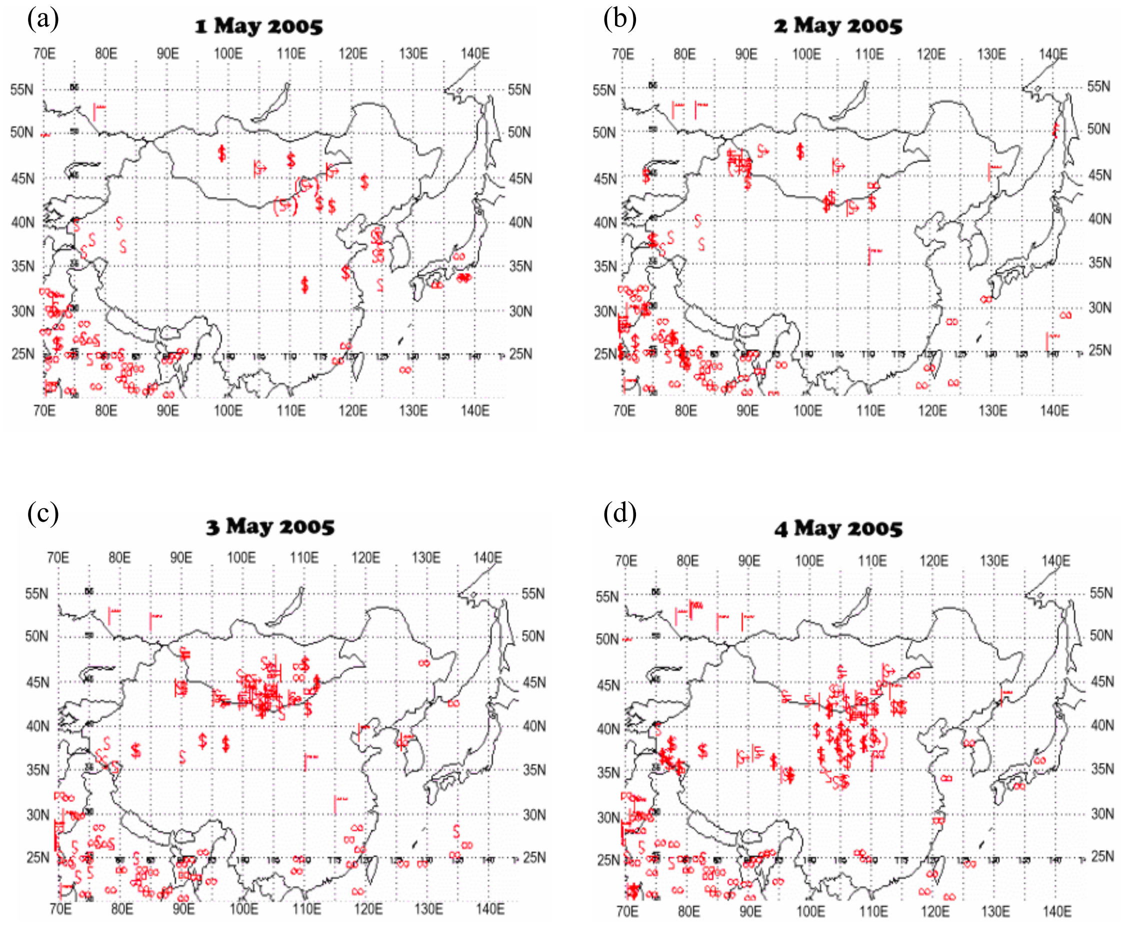

Fig. S1: Surface observation of dust storms happened on May 2-4, 2005. Symbols are explained in Table S1 and are WMO (World Meteorological Organization) standardized weather observation symbols. On May 1st, local dust storms associated with previous weather system were noted over deserts D_2 and D_4 (marked in Fig. 2). Then the intrusion of Siberia cold air mass forced dust storms over desert D_1 on May 2nd, later over deserts D_4, D_2 and D_3 on May 3rd and 4th. The sequence of occurrence and the eventually wide-spreading on May 4th followed with the movement of cold air mass marked as solid purple lines in Fig. S2.
