# Supplementary material for: Temperature Drops and the Onset of Severe Avian Influenza A H5N1 Virus Outbreaks
Source: PLoS One. 2007 Feb 7;2(2):e191. doi: 10.1371/journal.pone.0000191 (PMC1794318; doi:10.1371/journal.pone.0000191)
Supplement: Figure S2 — Locations of Altay mountains (M1), Tianshan Mountains(M2), Karakorum Mountains(M3), Kunlun Mountains(M4), Arjin Mountains(M5), Qilian Mountains(M6), Gurbantunggut desert(D1), Taklamakan desert(D2), Tsaidam desert(D3), Gobi desert(D4), Tsaidam Basin (B), Gangca city (O) and the Bird Mountain (△) at Qinghai Lake. Square mark (▭) indicates the area in Qinhgai, China where wild birds with H5N1 virus were reported in spring 2006. Solid purple lines indicate the movement of Siberia cold air mass which would trigger the development of dust storms. (0.19 MB PDF) [file pone.0000191.s002.pdf]

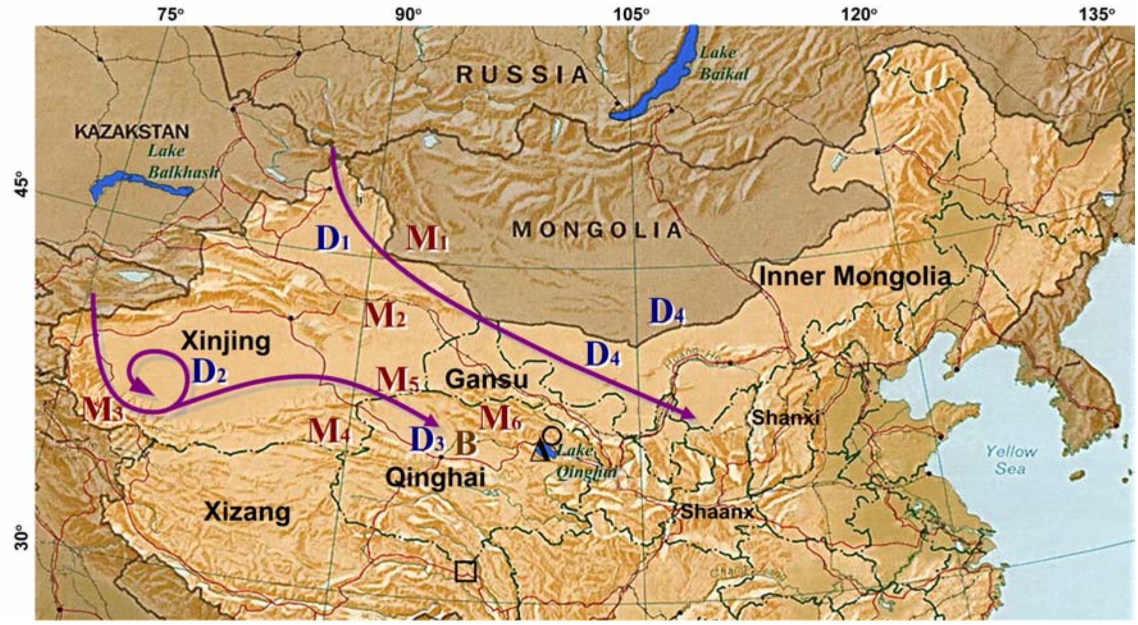

Figure S2: Locations of Altay mountains (M_1), Tianshan Mountains(M_2), Karakorum Mountains(M_3), Kunlun Mountains(M_4), Arjin Mountains(M_5), Qilian Mountains(M_6), Gurbantunggut desert(D_1), Taklamakan desert(D_2), Tsaidam desert(D_3), Gobi desert(D_4), Tsaidam Basin (B), Gangca city (O) and the Bird Mountain (△) at Qinghai Lake. Square mark (□) indicates the area in Qinhgai, China where wild birds with H5N1 virus were reported in spring 2006. Solid purple lines indicate the movement of Siberia cold air mass which would trigger the development of dust storms.
