# Supplementary material for: Temperature Drops and the Onset of Severe Avian Influenza A H5N1 Virus Outbreaks
Source: PLoS One. 2007 Feb 7;2(2):e191. doi: 10.1371/journal.pone.0000191 (PMC1794318; doi:10.1371/journal.pone.0000191)
Supplement: Figure S3 — Contour plots of sea level pressure, surface temperature and wind flow on selected day 0 of outbreak event I-a (2005/5/4) and event II-a (2005/7/19), II-b (2005/8/14), II-c (2005/9/28), II-d (2005/10/7), II-e (2005/10/10). (0.29 MB PDF) [file pone.0000191.s003.pdf]

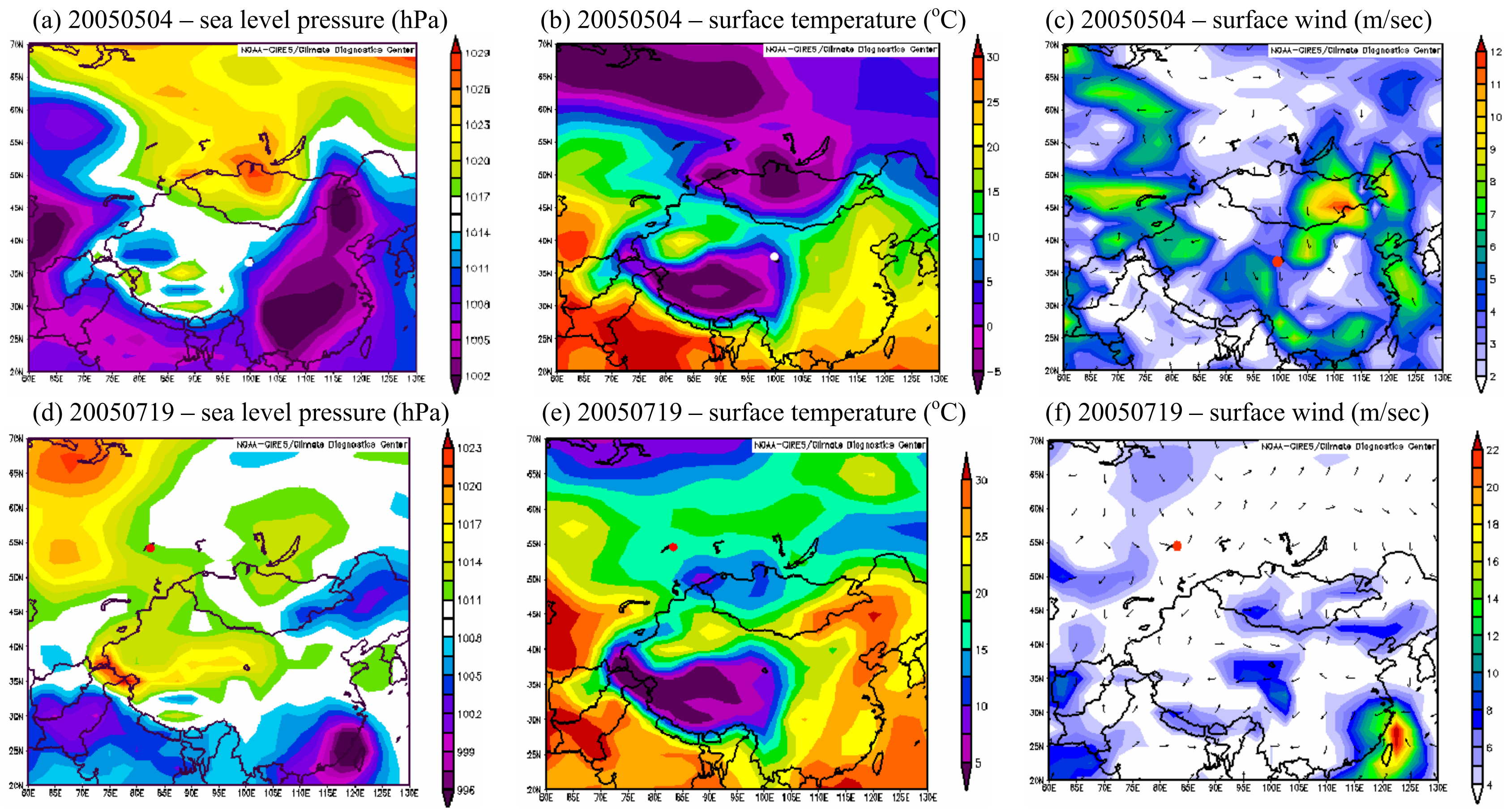

Figure S3: Contour plots of sea level pressure, surface temperature and wind flow on selected day 0 of outbreak event I-a (2005/5/4) and event II-a (2005/7/19), II-b (2005/8/14), II-c (2005/9/28), II-d (2005/10/7), II-e (2005/10/10). Each plot is downloaded from NOAA CDC Interactive Plotting and Analysis Pages (http://www.cdc.noaa.gov/ Composites/Day/) using NCEP reanalysis data. In each figure, either a white dot or a red dot is marked to indicate the area where avian influenza broke out.

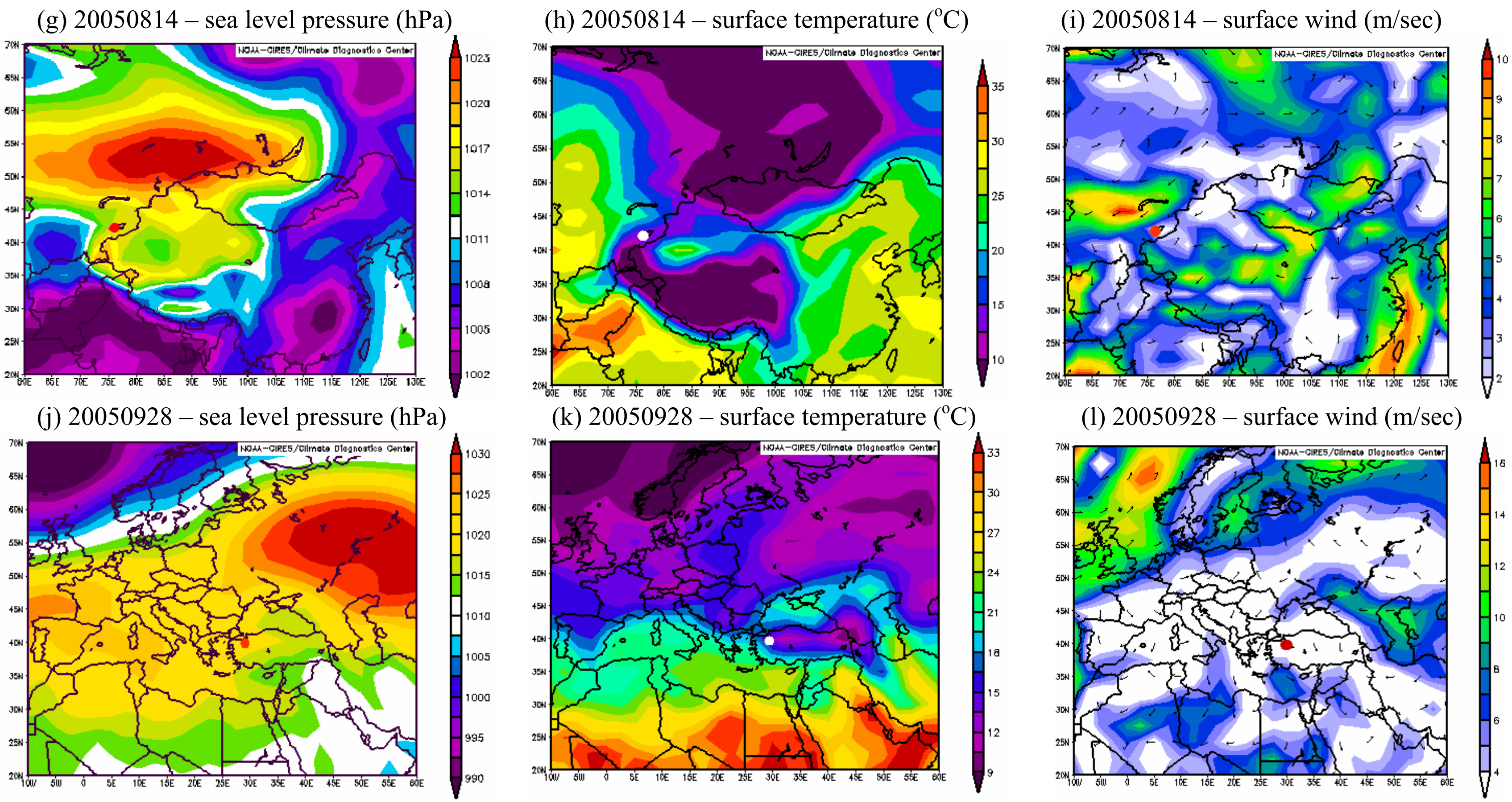

Figure S3: (continued)

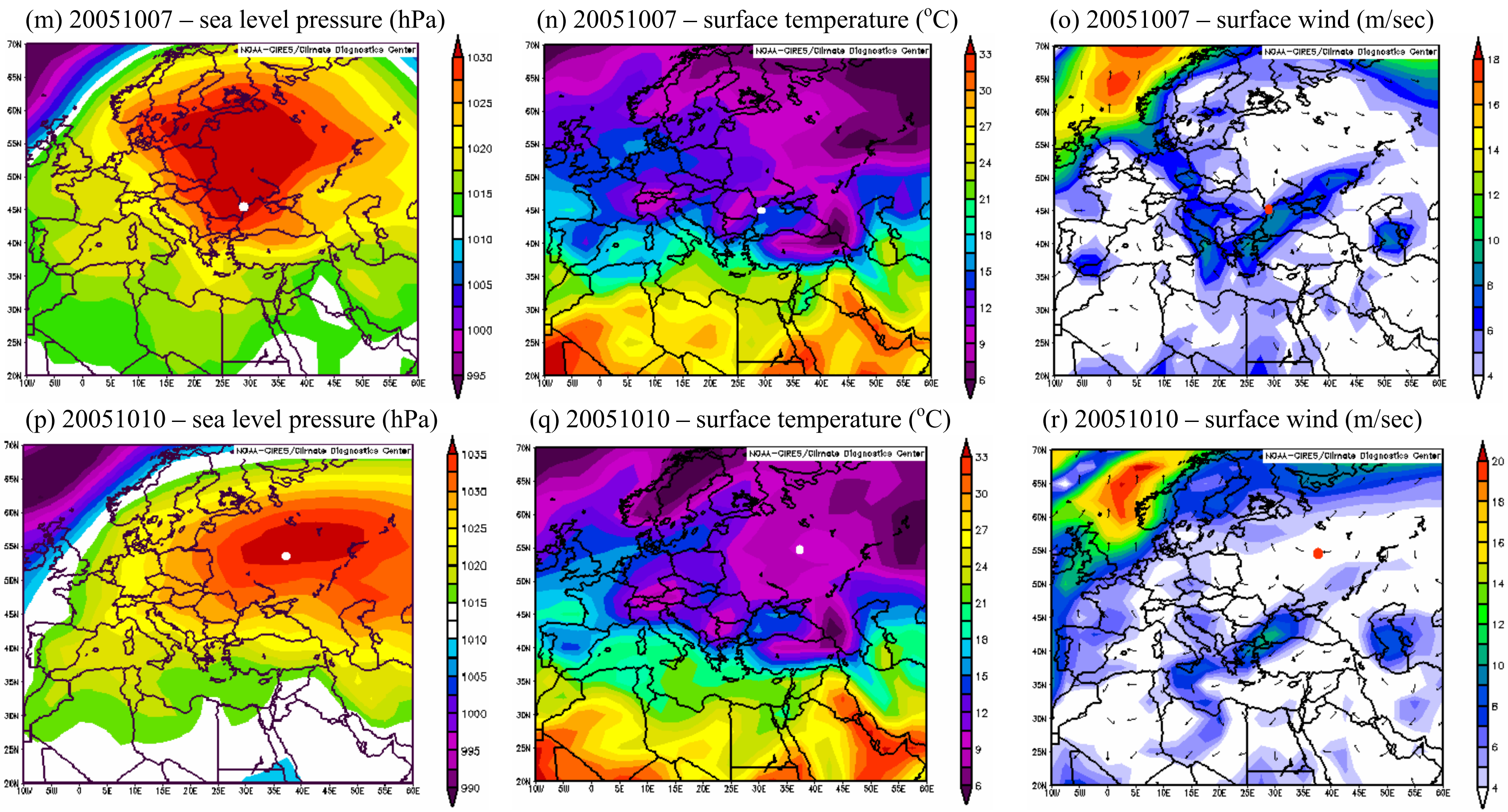

Figure S3: (continued)
