# Supplementary material for: Temperature Drops and the Onset of Severe Avian Influenza A H5N1 Virus Outbreaks
Source: PLoS One. 2007 Feb 7;2(2):e191. doi: 10.1371/journal.pone.0000191 (PMC1794318; doi:10.1371/journal.pone.0000191)
Supplement: Figure S4 — Contour plots of sea level pressure, surface temperature and wind flow on selected day 0 of outbreak event III-a (2005/10/13), III-b (2005/10/21), III-c (2005/11/5), III-d (2005/11/8). (0.18 MB PDF) [file pone.0000191.s004.pdf]

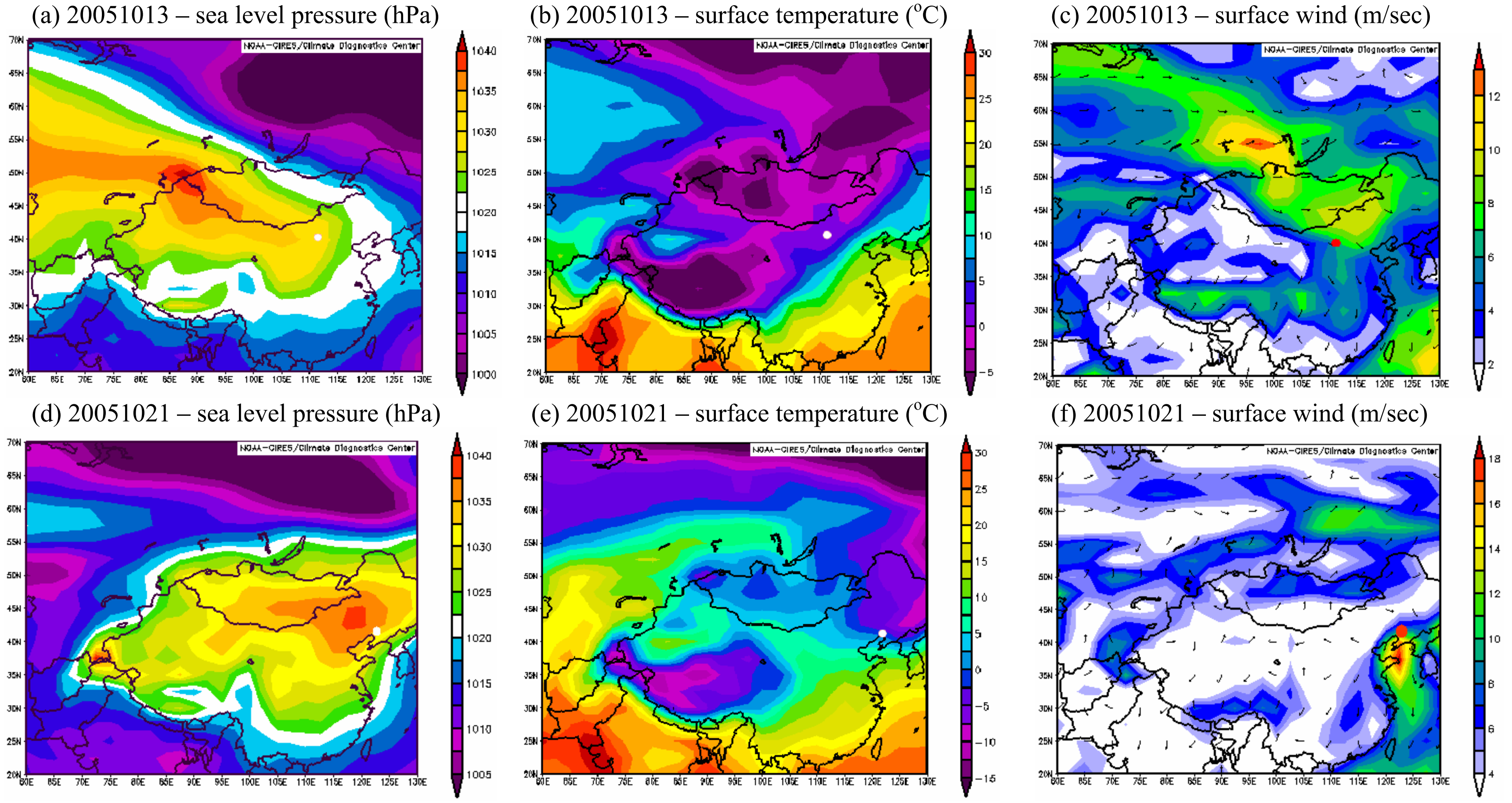

Figure S4: Contour plots of sea level pressure, surface temperature and wind flow on selected day 0 of outbreak event III-a (2005/10/13), III-b (2005/10/21), III-c (2005/11/5), III-d (2005/11/8). Each plot is downloaded from NOAA CDC Interactive Plotting and Analysis Pages (http://www.cdc.noaa.gov/Composites/Day/) using NCEP reanalysis data. In each figure, a white dot or a red dot is marked to indicate the area where avian influenza broke out.

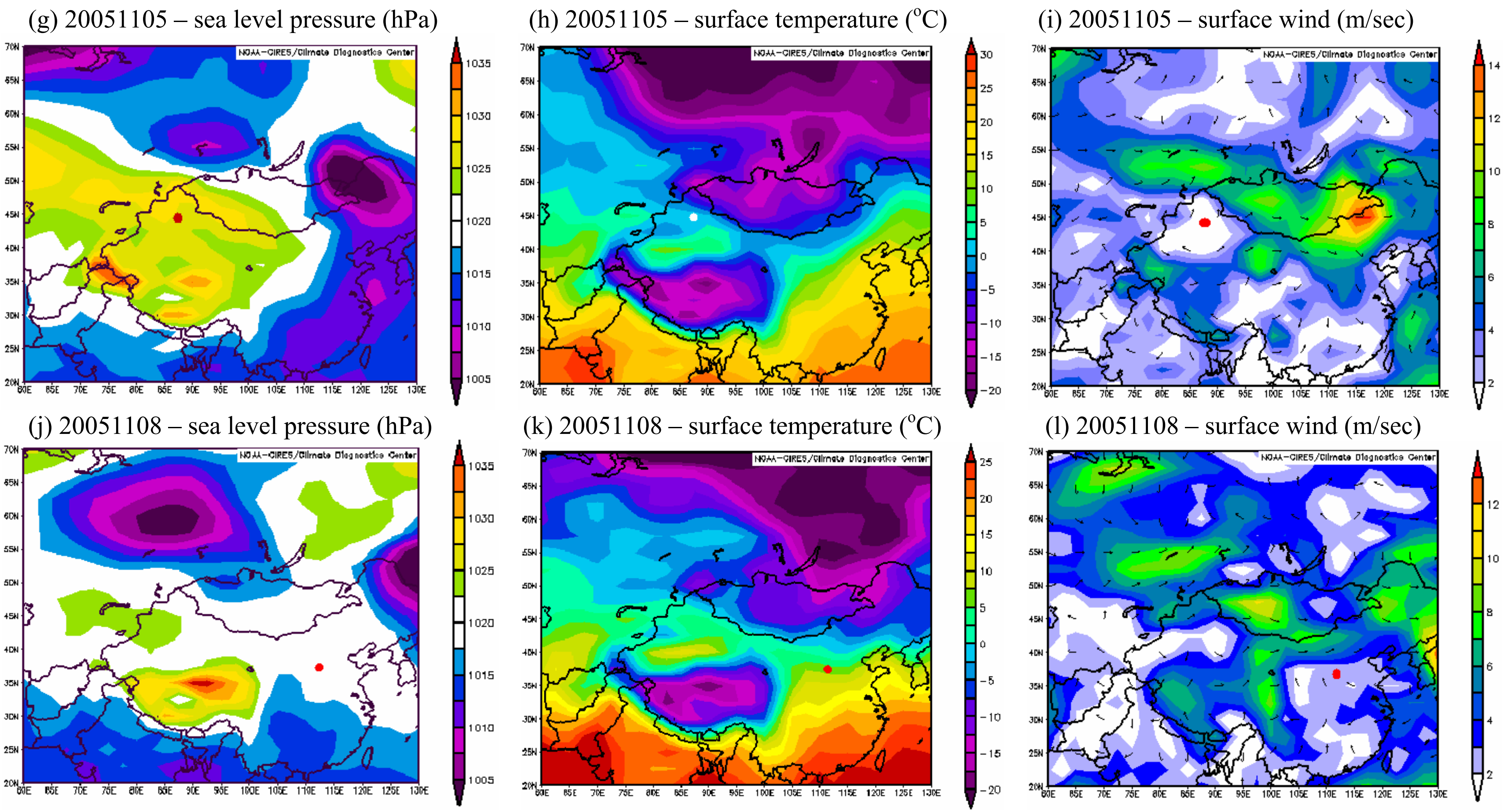

Figure S4: (Continued)
