# Supplementary material for: Temperature Drops and the Onset of Severe Avian Influenza A H5N1 Virus Outbreaks
Source: PLoS One. 2007 Feb 7;2(2):e191. doi: 10.1371/journal.pone.0000191 (PMC1794318; doi:10.1371/journal.pone.0000191)
Supplement: Figure S6 — Contour plots of sea level pressure, surface temperature and wind flow on selected day 0 of outbreak event V-a (2006/1/26), V-b (2006/2/6), V-c (2006/2/12), V-d (2006/2/27), V-e (2006/3/8). (0.27 MB PDF) [file pone.0000191.s006.pdf]

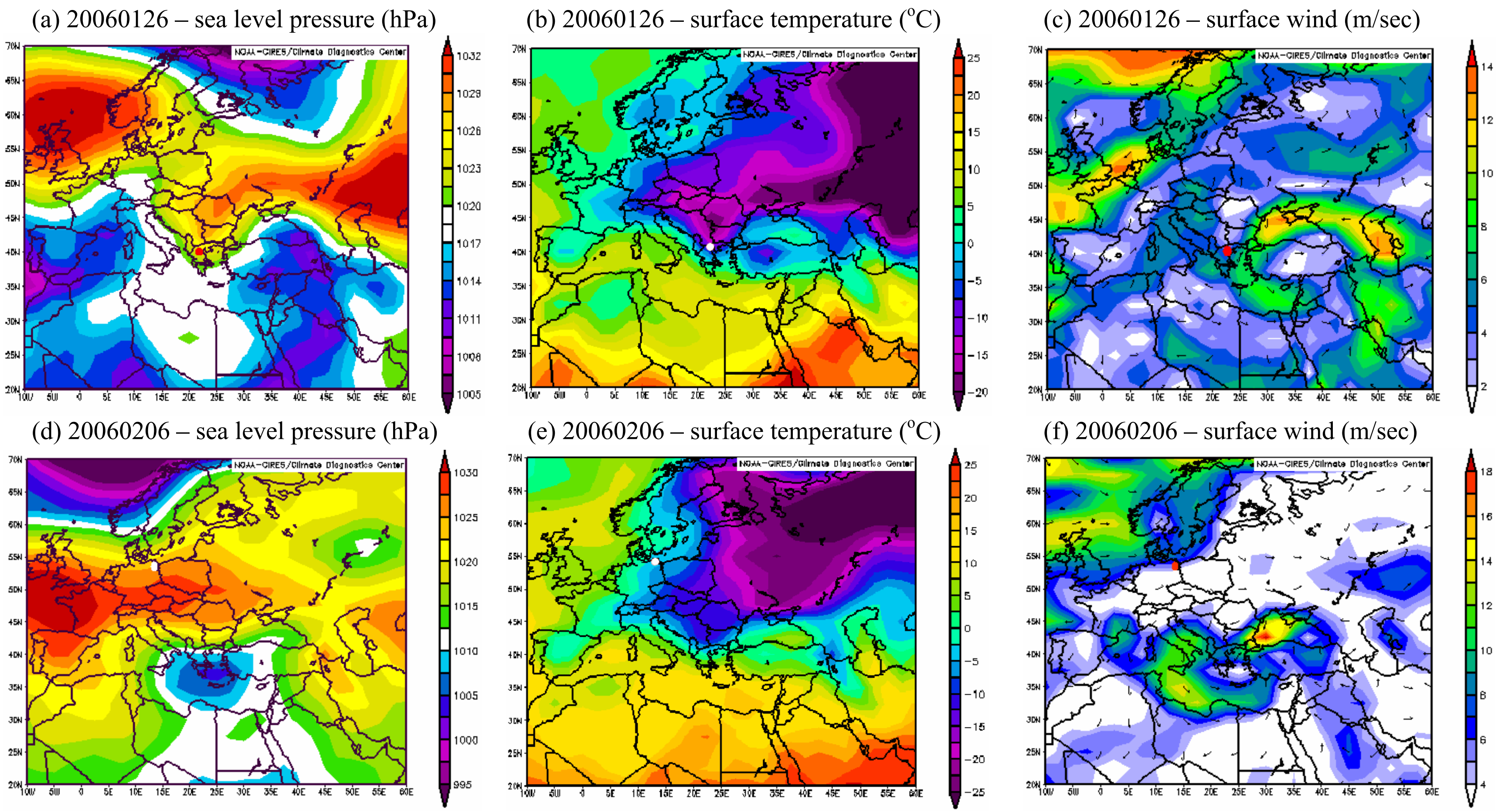

Figure S6: Contour plots of sea level pressure, surface temperature and wind flow on selected day 0 of outbreak event V-a (2006/1/26), V-b (2006/2/6), V-c (2006/2/12), V-d (2006/2/27), V-e (2006/3/8). Each plot is downloaded from NOAA CDC Interactive Plotting and Analysis Pages (http://www.cdc.noaa.gov/Composites/Day/) using NCEP reanalysis data. In each figure, a white dot or a red dot is marked to indicate the area where avian influenza broke out.

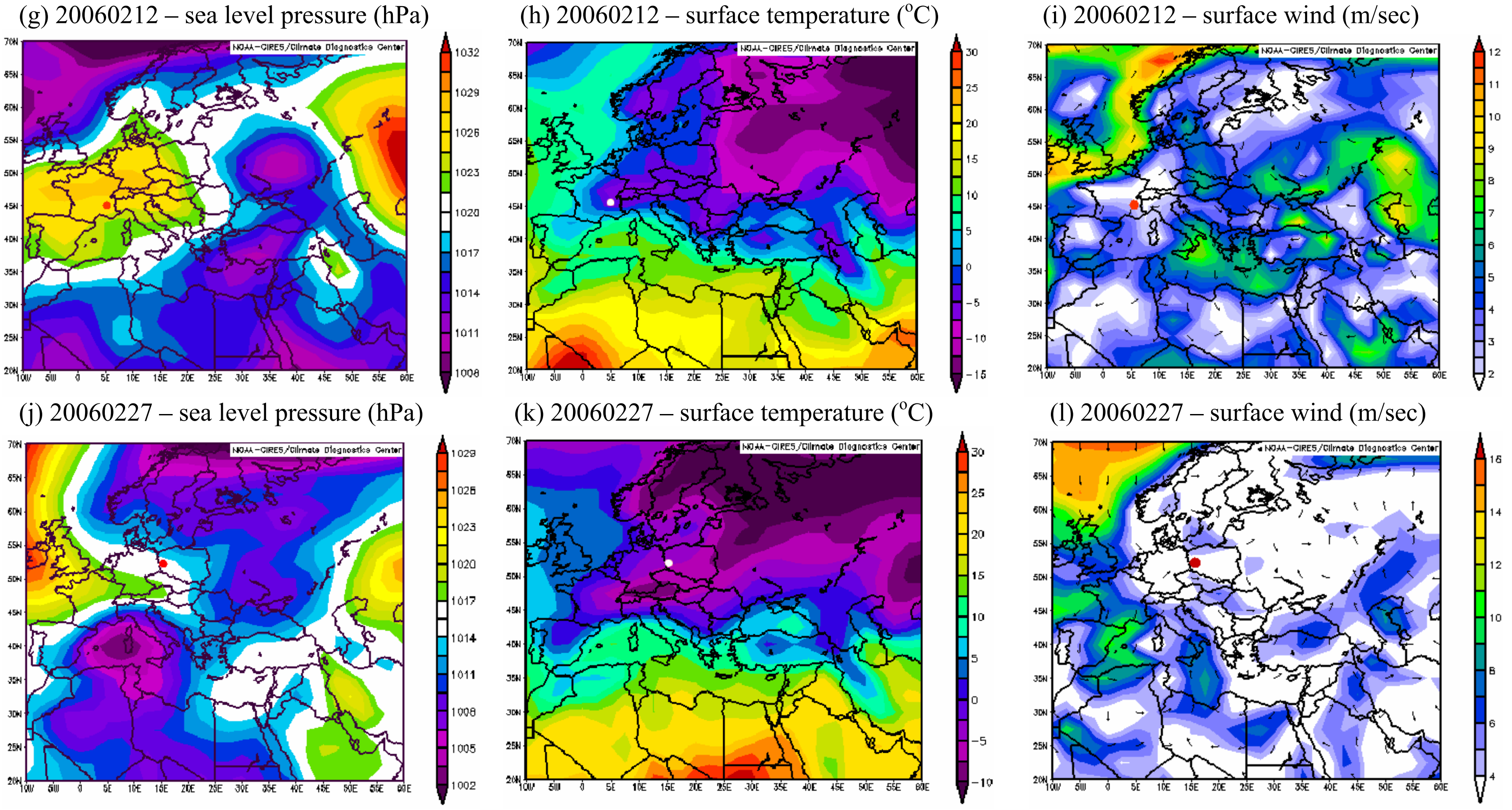

Figure S6: (continued)

(m) 20060308 – sea level pressure (hPa) (n) 20060308 – surface temperature (^{o}C) (o) 20060308 – surface wind (m/sec)

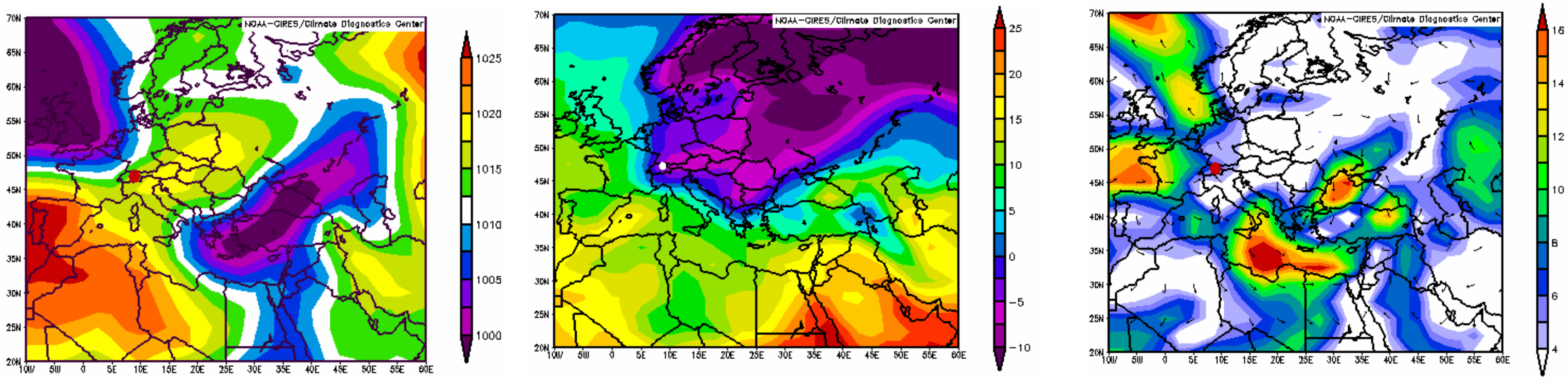

Figure S6: (continued)
