# Supplementary material for: Temperature Drops and the Onset of Severe Avian Influenza A H5N1 Virus Outbreaks
Source: PLoS One. 2007 Feb 7;2(2):e191. doi: 10.1371/journal.pone.0000191 (PMC1794318; doi:10.1371/journal.pone.0000191)
Supplement: Figure S7 — Contour plots of sea level pressure, surface temperature and wind flow on selected day 0 of outbreak event VI-a (2006/2/18), VI-b,c (2006/3/11) and VII-a (2006/4/15), VII-b (2006/5/21), VII-c (2006/5/26), VII-d (2006/6/15). Each plot is downloaded from NOAA CDC Interactive Plotting and Analysis Pages (http://www.cdc.noaa.gov/Composites/Day/) using NCEP reanalysis data. In each figure, a white dot or a red dot is marked to indicate the area where avian influenza broke out. (0.30 MB PDF) [file pone.0000191.s007.pdf]

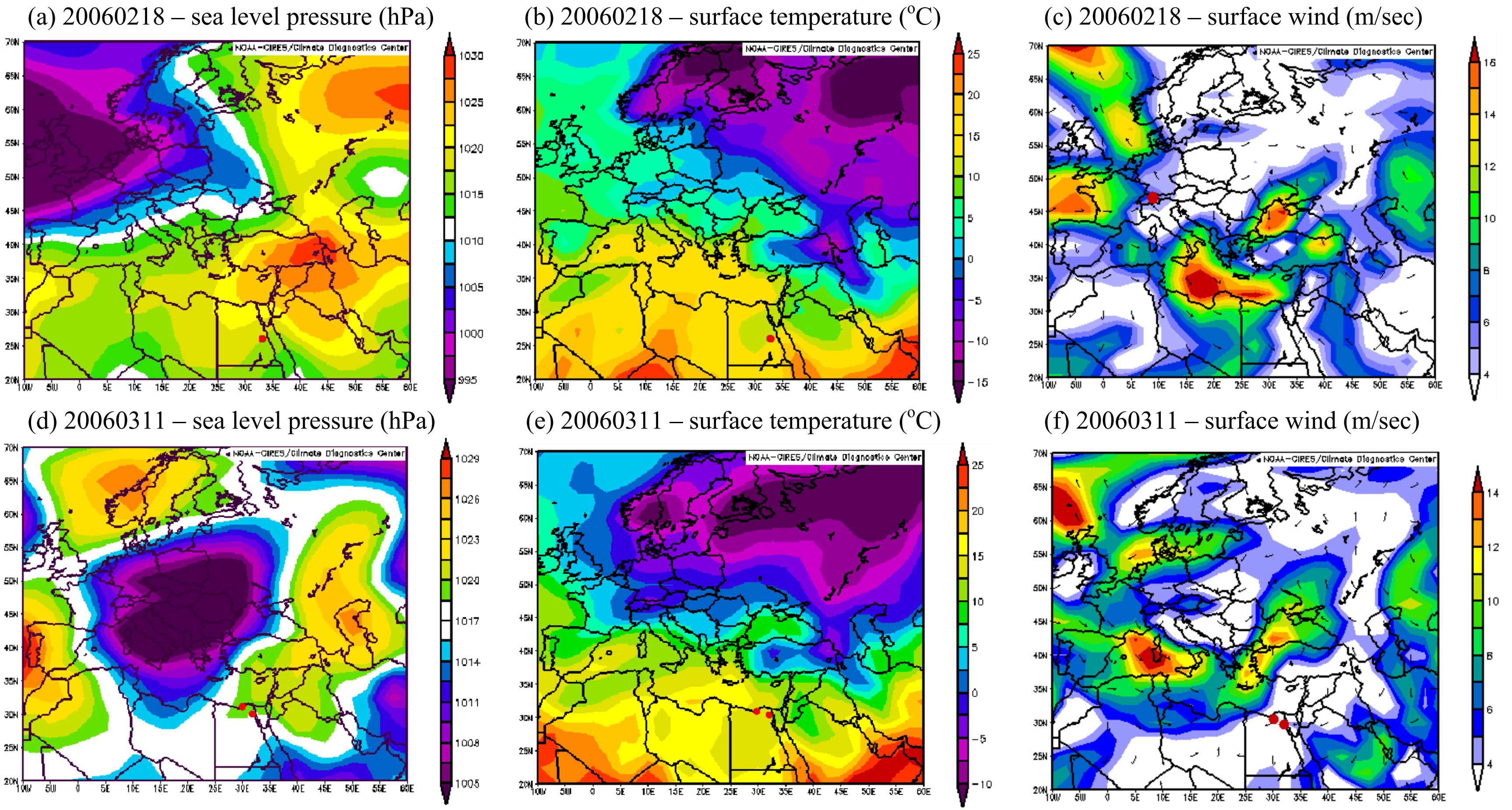

Figure S7: Contour plots of sea level pressure, surface temperature and wind flow on selected day 0 of outbreak event VI-a (2006/2/18), VI-b,c (2006/3/11) and VII-a (2006/4/15), VII-b (2006/5/21), VII-c (2006/5/26), VII-d (2006/6/15). Each plot is downloaded from NOAA CDC Interactive Plotting and Analysis Pages (http://www.cdc.noaa.gov/Composites/Day/) using NCEP reanalysis data. In each figure, a white dot or a red dot is marked to indicate the area where avian influenza broke out.

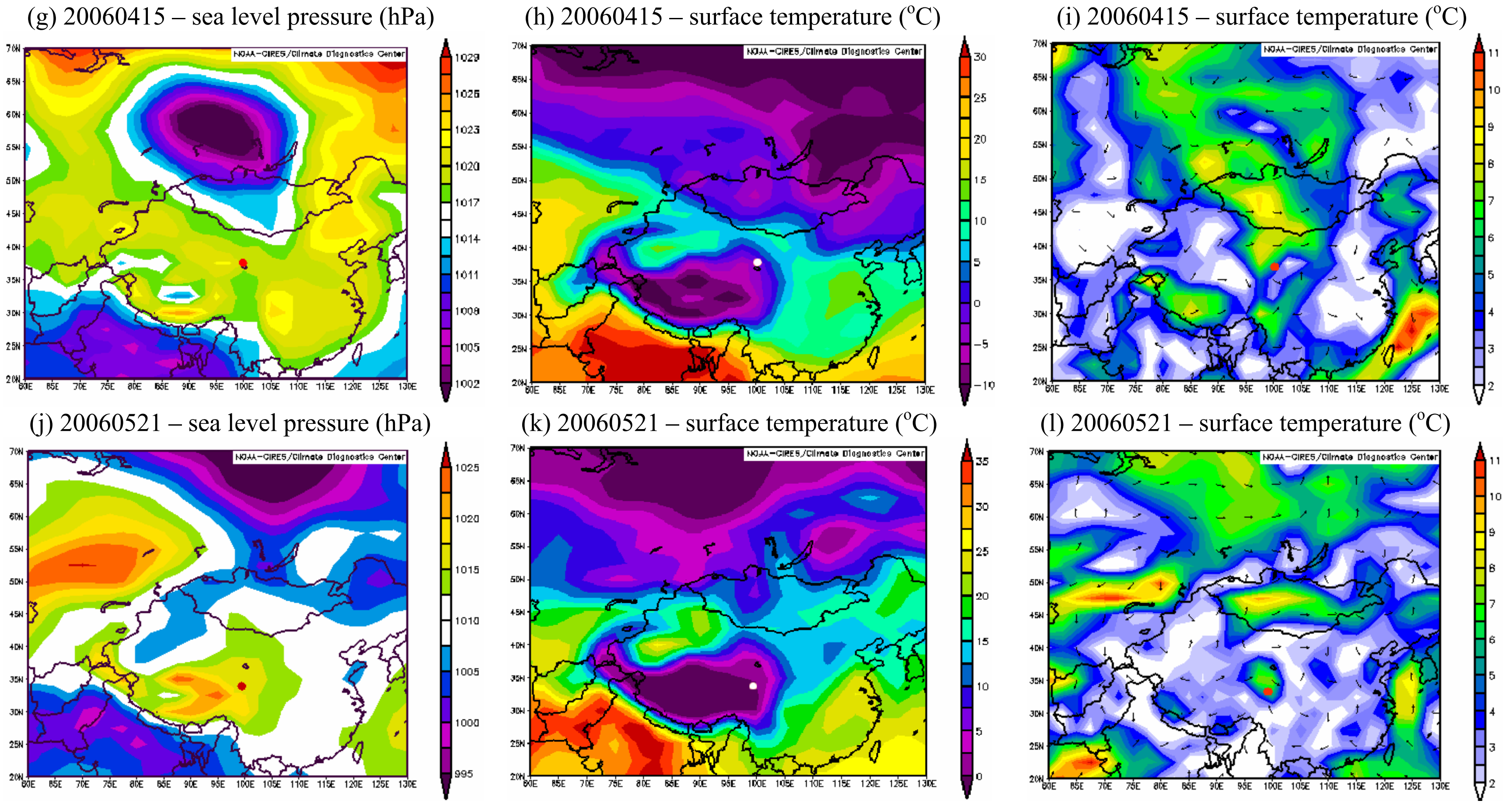

Figure S7: (continued)

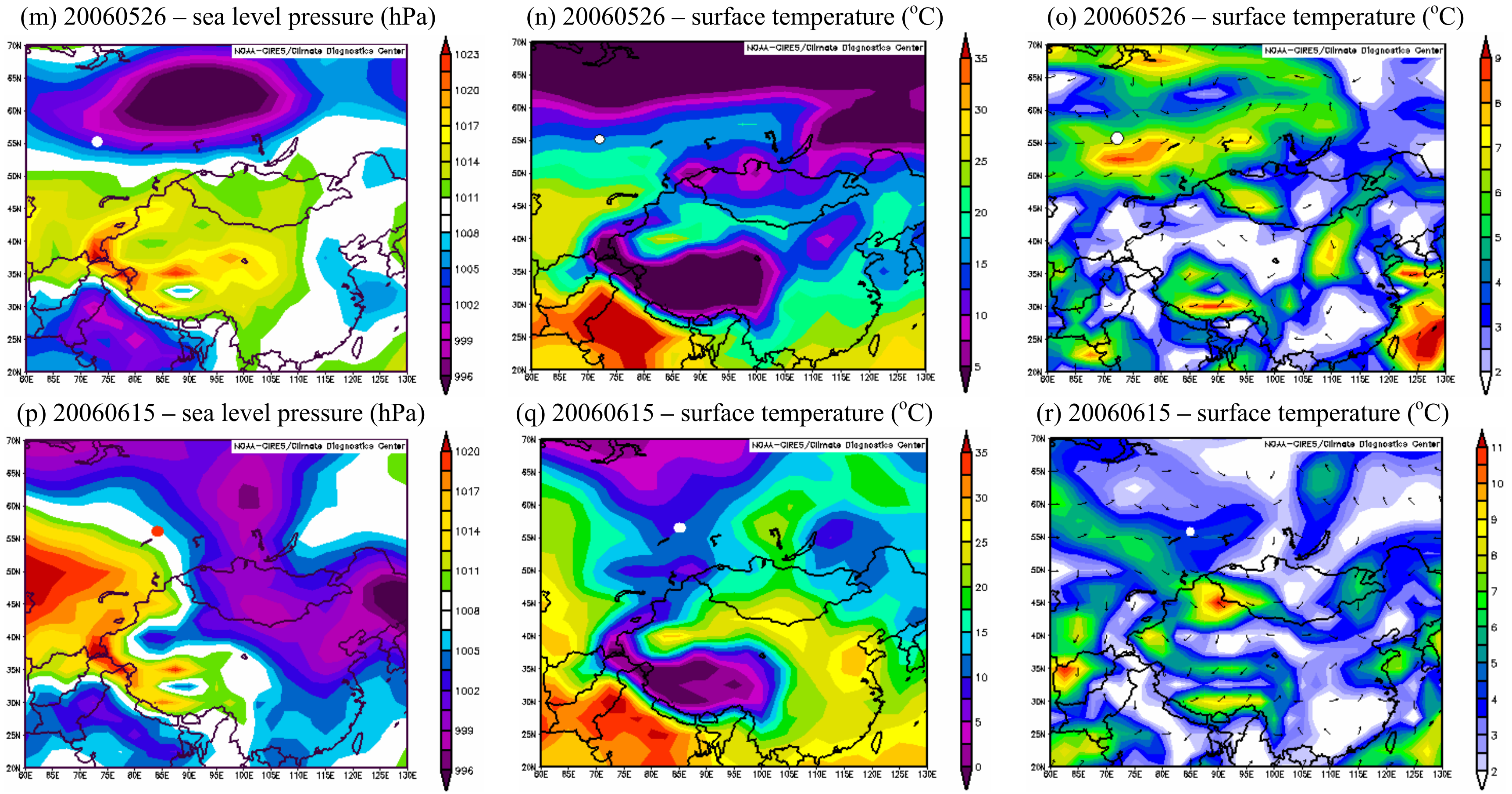

Figure S7: (continued)
