# Supplementary material for: Temperature Drops and the Onset of Severe Avian Influenza A H5N1 Virus Outbreaks
Source: PLoS One. 2007 Feb 7;2(2):e191. doi: 10.1371/journal.pone.0000191 (PMC1794318; doi:10.1371/journal.pone.0000191)
Supplement: Table S1 — Meaning of weather symbols shown in Fig. S1. (0.10 MB PDF) [file pone.0000191.s008.pdf]

Table S1: Meaning of weather symbols shown in Fig. S1.

code	symbol	Description
04	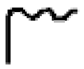	Visibility reduced by smoke haze
05	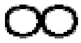	Haze
06	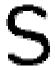	Widespread dust in suspension in the air, not raised by wind at or near the station at the time of observation.
07	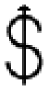	Dust or sand raised by the wind at or near the station at the time of the observation, but no well-developed dust whirl(s), and no sandstorm seen: or, in the case of ships, blowing spray at the station
08	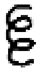	Well developed dust whirl(s) or sand whirl(s) seen at or near the station during the preceding hour or at the time of observation, but no dust storm or sandstorm
09	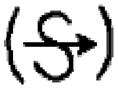	Well developed dust whirl(s) or sand whirl(s) seen at or near the station during the preceding hour or at the time of observation, but no dust storm or sandstorm
30	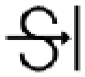	Slight or moderate dust storm or sand storm has decreased during past hour
31	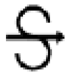	Slight or moderate dust storm or sand storm no appreciable change during past hour
32	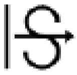	Slight or moderate dust storm or sand storm has increased during past hour
33	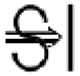	Severe dust storm or sand storm has decreased during past hour
34	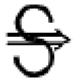	Slight or moderate dust storm or sand storm no appreciable change during past hour
35	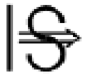	Slight or moderate durst storm or sand storm has increased during past hour
98	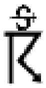	Thunderstorm combined with dust storm or sandstorm at time of observation
